# Knowledge of pulse oximetry, indications for oxygen therapy, and integrated management of childhood illness among health care workers in Nigerian primary and secondary health facilities: a cross-sectional survey

**DOI:** 10.3389/fpubh.2026.1789259

**Published:** 2026-07-08

**Authors:** Ayobami A. Bakare, Olumide Ebenezer Olufayo, Obioma Uchendu, James Yewande, Julius Salako, Omotayo Olojede, Ayodamola Bakare, Carina King, Adegoke G. Falade

**Affiliations:** 1 Department of Community Medicine, University College Hospital, Ibadan, Nigeria; 2 Department of Global Public Health, Karolinska Institutet, Solna, Sweden; 3 Oxygen for Life Initiative, Ibadan, Nigeria; 4 Department of Community Medicine, University of Ibadan, Ibadan, Nigeria; 5 School of Health Sciences, Ulster University, Derry, United Kingdom; 6 Department of Paediatrics, University College Hospital, Ibadan, Nigeria; 7 Department of Paediatrics, University of Ibadan, Ibadan, Nigeria

**Keywords:** child survival, children, health system, IMCI, knowledge, oxygen, service delivery

## Abstract

**Background:**

The WHO’s Integrated Management of Childhood Illness (IMCI) strategy aims to reduce deaths among children under the age of five. Pulse oximetry, a simple non-invasive method for measuring peripheral oxygen saturation - an indicator of severe illness, can enhance IMCI implementation. In Nigeria, despite efforts to incorporate pulse oximetry into IMCI, its adoption is sub-optimal. We assessed healthcare workers’ (HCWs) knowledge of pulse oximetry and IMCI in primary and secondary facilities to identify opportunities for improvement.

**Methods:**

We conducted a cross-sectional study in 54 primary health facilities and 8 secondary hospitals purposively selected across five different states in Nigeria: Oyo, Lagos, Rivers, Kano and Jigawa. The data was collected between 6 March 2025 and 30 June 2025 through an interviewer-assisted questionnaire, which was developed from the national oxygen policy and WHO guidelines. We used descriptive statistics to summarise our findings. Pulse oximetry knowledge scores were dichotomised, and we used bivariate logistic regression to assess associations between HCWs’ cadre and pulse oximetry knowledge. For IMCI knowledge, we reported mean scores and used one-way analysis of variance to assess associations between mean scores and the HCWs’ cadre.

**Results:**

Of the 463 eligible HCWs recruited, 75.4% were female, 52.3% were community health workers (CHWs), and 73.8 and 26.2% were employed by the government and facility, respectively. Overall, 49.7% knew that pulse oximetry should be performed for all presenting patients, with substantial variation across cadres (39.4% among CHWs, 74.4% among doctors, 52.9% among nurses). Doctors had 3.1 times higher odds of having good pulse oximetry knowledge compared to other HCWs (95% CI: 1.17–8.04). Knowledge of IMCI general danger signs was mixed with convulsion (93.5%), lethargy (80.9%) and loss of consciousness (84.6%) being mostly reported as general danger signs compared to vomiting everything and being unable to eat/drink. Less than 10% of the HCWs knew the four main symptoms for assessment in children aged 2–59 months.

**Conclusion:**

IMCI Knowledge was generally poor across all HCW cadres, but pulse oximetry knowledge varied substantially. Strengthening pre-service and in-service IMCI and pulse oximetry training, particularly for CHWs and nurses is essential to improve childhood illness management and oxygen therapy practices in Nigeria.

## Background

Since its launch in 1995, the WHO’s Integrated Management of Childhood Illness (IMCI) strategy has been adopted by over 100 countries, including Nigeria, to reduce childhood mortality and promote healthy development (1, 2). Despite this, its implementation in Nigeria has been suboptimal (3–5). Nigeria accounts for the largest (approximately 17.6%) of global under-five deaths (6, 7), many of which are preventable through effective IMCI delivery (7). Understanding healthcare workers’ (HCWs) knowledge of IMCI is an essential step in addressing current implementation gaps and strengthening child survival interventions.

Hypoxaemia, defined as a low blood oxygen saturation (SpO_2_) is a life-threatening complication of many clinical conditions, and it requires prompt identification and treatment. Pulse oximetry is a non-invasive method to measure peripheral oxygen saturation levels, and it is the WHO-recommended method for detecting hypoxaemia in low-resource settings (8). Its integration into the IMCI algorithm is crucial for improving the triage and management of severely ill children through prompt hypoxaemia detection. Recognising this, the Nigerian Federal Ministry of Health in 2017 adopted pulse oximetry as the fifth vital sign (9). However, this has not translated into increased pulse oximetry use, as studies have shown that just 1 out of every 10 children presenting at primary health facilities had pulse oximetry readings despite the availability of pulse oximeters Limited in Nigeria (10, 11).

Improving pulse oximetry practice remains a national and global priority. The Lancet Global Health Commission on Medical Oxygen includes pulse oximetry coverage as one of the core indicators of oxygen system readiness (12). Similarly, the revised 2023 Nigerian National Strategy for the Scale-up of Medical Oxygen set a target of achieving at least 80% pulse oximetry coverage in health facilities by 2027 (13). Yet, facility audits in two States indicated that pulse oximetry coverage is low in secondary hospitals and negligible in primary health centers (PHCs) (14). To reduce this policy-practice gap in pulse oximetry adoption in Nigeria, local evidence on key barriers to uptake, like HCW knowledge is essential.

However, available evidence in Nigeria on HCWs’ knowledge of pulse oximetry and IMCI is mainly normative (15, 16), rather than an assessment of knowledge that informs their real-world decision-making in a clinical context. Other studies have been limited to a particular region (17–19), or limited to single facilities (20), thus limiting their generalizability across the Nigerian context. There is a lack of a multi-state, facility-level assessment that objectively measures the knowledge guiding clinical decision-making for both IMCI and pulse oximetry. To fill this gap and inform the ongoing national efforts to incorporate pulse oximetry into IMCI training modules, we aimed to assess the knowledge of HCWs in PHCs and secondary hospitals about pulse oximetry and IMCI, to identify training and knowledge gaps and inform strategies for improving adherence to national IMCI and oxygen protocols.

## Materials and methods

### Study design

We conducted a cross-sectional study in 54 primary health facilities and 8 secondary hospitals across five States in Nigeria: Oyo, Lagos, Rivers, Kano and Jigawa. The data was collected between 6 March 2025 and 30 June 2025. The states were purposefully selected based on their geographical variation, feasibility of data collection and to improve the generalizability of the study within Nigerian context and capture nuanced differences across settings.

### Settings

We included Oyo and Lagos state in southwest Nigeria, Jigawa and Kano state in Northwestern Nigeria, and Rivers state in South–south Nigeria (Appendix 1). Under-five mortality differs across the states, ranging from 50 deaths per 1,000 live births in Lagos to 174 deaths per 1,000 live births in Jigawa (21). We purposively selected 1–5 local government areas (LGAs) in each state based on the following criteria: accessibility, location, and feasibility of data collection. In each selected LGA, we selected health facilities from rural and urban settings to ensure geographical representation. Additionally, the team included facilities with different workload levels to capture a diverse range of service delivery contexts.

### Study population

The population of interest is HCWs in primary and secondary health facilities. We included HCWs from different cadres known to provide medical or clinical services in Nigeria, who are involved in patient clinical assessment. HCWs were conveniently sampled.

### Sample size calculation

The sample size was calculated as illustrated below using the formula for single population (22): $N=\frac{Z^{2}p(1-p)}{d^{2.}}$

Assuming a 95% confidence level ( Z=1.96 ), a prevalence of good knowledge of IMCI of 45.8% ( p=0.458 ) (17), and a desired level of precision of 5% ( d=0.05 ). This would give an initial sample size of 381. A finite population correction was applied, given an estimated total of 2,500 healthcare workers across the selected LGAs:


$$\mathrm{Nf}=\frac{381}{1+\frac{381-1}{2500}}=331$$

Adjusting for a 10% non-response rate, the final sample size was: $N=\frac{331}{0.9}=368$

### Data collection

Data was collected through an interviewer-assisted questionnaire, which was developed from the national oxygen policy and the WHO IMCI guidelines (9, 23). We obtained data on HCWs’ socio-demographic details (age, gender, employment status), training experience, and pulse oximetry and IMCI knowledge. Knowledge questions on pulse oximetry assessed HCWs’ understanding of its use, when to use a pulse oximeter, indications for oxygen therapy and normal oxygen saturation. For IMCI, we assessed HCWs’ knowledge of general danger signs, diagnosis and treatment of common childhood illnesses, and knowledge of the four major IMCI symptoms.

Data collectors were nurses (*n* = 10) and trained research assistants with bachelor’s degrees (*n* = 6) with past experience in data collection for similar projects (5, 11, 24). Each data collector was assigned 4–5 health facilities and visited them on different days, based on a random allocation conducted by AAB and JS. On each visit, the data collector obtained data from all consenting healthcare workers who met the inclusion criteria. Although repeat visits were conducted throughout the study period to ensure full coverage, each HCW completed the questionnaire only once. Data was collected through CommCare using an encrypted Android-based mobile phone. Data collectors documented all HCWs approached using mobile tablets. Upon obtaining informed consent, the tablet was handed to each consenting HCW to self-complete the questionnaire.

### Data management

We used descriptive statistics to summarise our findings. For pulse oximetry, one point was awarded for each correct response (Appendix 2), resulting in a maximum total knowledge score of four. Based on the national oxygen policy and the revised 2023 national strategy for the scale up of medical oxygen in Nigeria (9, 13). Scores of less than four were classified as indicating poor knowledge, whereas a score of four was classified as good knowledge. We used bivariate logistic regression to assess associations between HCWs’ cadre and pulse oximetry knowledge. We adjusted for state, given past oxygen-related intervention in the selected states (25–27), and contextual differences in health system and socioeconomic differences. For IMCI, we assessed knowledge of danger signs using eight questions and knowledge of IMCI diagnosis and treatment using seven questions with eleven correct answers. Each correct answer was scored as one point, and domain scores were summed to obtain an overall IMCI knowledge score. We compared the mean knowledge scores among HCWs using the one-way analysis of variance.

### Ethical consideration

The Helsinki Declaration and the Nigerian National Code of Health Research Ethics were followed in this investigation. Ethical approval was obtained from the relevant ethics authorities, including the University of Ibadan/University College Hospital (Ref: UI/EC/24/0607), Jigawa State Government (ref: JGHREC/2024/0064), Kano State (ref: NHREC/17/03/2018), Lagos State Government (Ref: LREC/06/10/2587), Oyo State Ministry of Health (ref: AD 13/479/873B), and Rivers State (ref: RSHMB/RSHREC/2024/089). Following the approvals, we visited all selected facilities to inform the facility management and to seek their approval. All HCWs involved in this study gave verbal consent before taking part, and they were given the chance to read the informed consent form. Participants were specifically informed that participation was voluntary and that the information gathered would only be utilised for research purposes.

## Results

**Figure 1:**
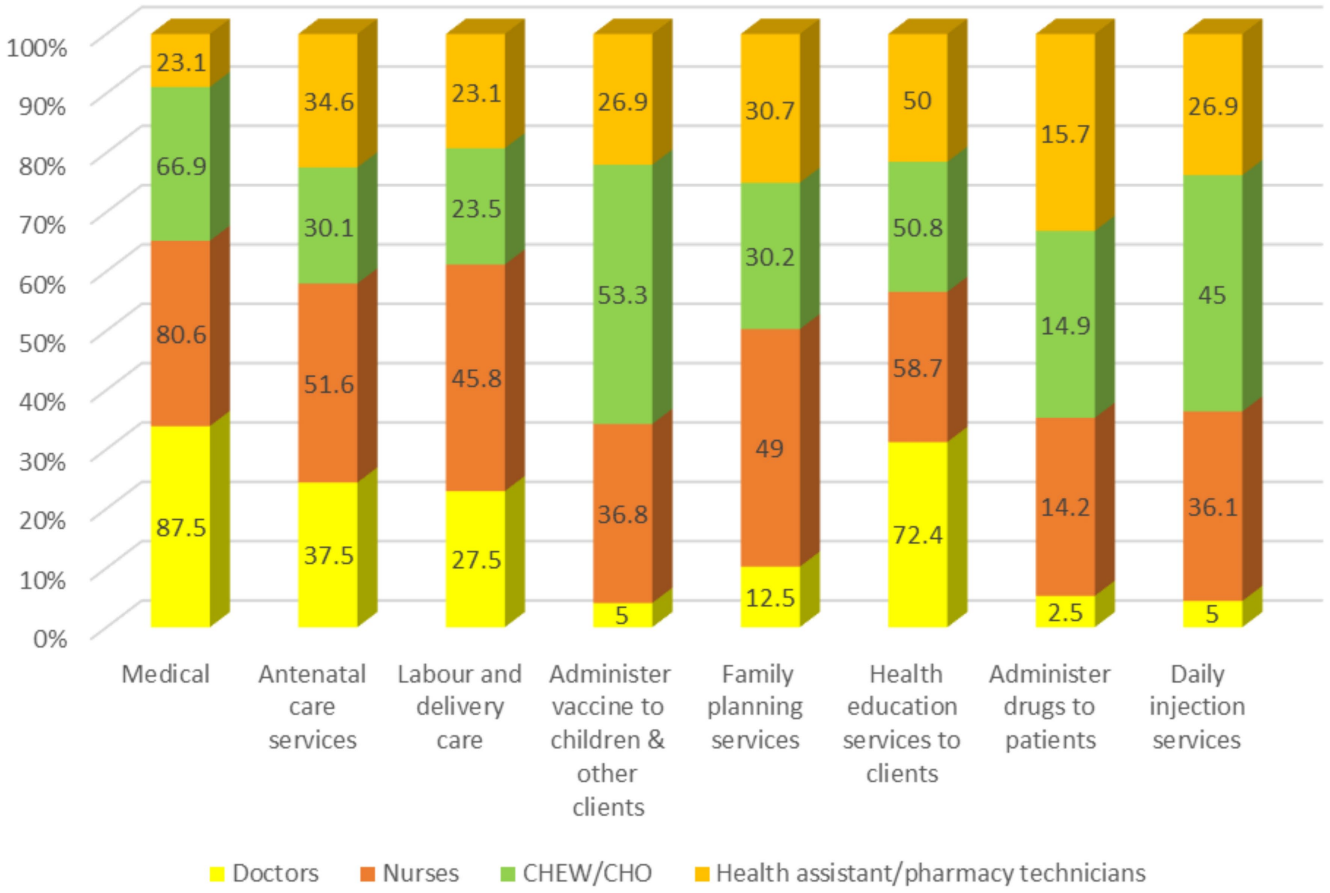
Roles of healthcare workers in primary health facilities.

From 714 HCWs approached, we included 463 respondents, of whom 75.4% were female, 52.3% were community health workers (CHWs), and 73.8% were employed by the government (Appendices 3, 4). Job duties varied across the different cadres: medical services were mostly provided by doctors (87.5%), nurses (80.6%), and CHWs (66.9%), while vaccination services were mainly delivered by CHWs (53.3%), nurses (36.8%), and health assistants or pharmacy technicians (26.9%) (Figure 1). Regarding training received, training on infection prevention and control was commonly reported across all cadres (doctors, 80.0%; nurses, 78.1%; CHWs, 68.6%; and health assistants/pharmacy technicians, 57.7%). Training on emergency triage assessment and treatment (ETAT) (doctors, 42.5%; nurses, 40.6%;, CHWs, 37.2% and health assistants/pharmacy technicians, 30.7%) and Integrated Community Case Management (iCCM) (doctors, 27.5%; nurses, 35.5%; CHWs, 42.9%; and health assistants/pharmacy technicians, 15.4%) were less commonly reported among HCWs (Appendix 5).

### Pulse oximetry knowledge

Approximately half of the respondents (49.7%) knew that pulse oximetry should be performed for any patient presenting to the health facility, although this varied by cadre—39.4% among CHWs compared with 74.4% among doctors and 52.9% among nurses (Table 1). A similar pattern was observed regarding knowledge of normal oxygen saturation among patients, with 36.5% of CHWs correctly selecting 95–100% compared to 76.9% among doctors and 59.6% among nurses. However, knowledge of the WHO-recommended oxygen saturation threshold for initiating oxygen therapy was low across all cadres (CHWs, 27.1%, doctors, 30.7% and nurses, 29.1%).

**Table 1:** Pulse oximetry knowledge among healthcare workers in Nigeria.

	Doctors (*N* = 40) Frequency (%)	Nurses (*N* = 155) Frequency (%)	CHEW/CHO (*N* = 242) Frequency (%)	Others (*N* = 26) Frequency (%)	Total (463)
Know a pulse oximeter	39/40 (97.5)	151/155 (97.4)	170/242 (70.2)	16/26 (61.5)	376/463 (81.2)
A pulse oximeter is used for measuring oxygen saturation levels (Multiple responses allowed)					
Blood pressure	1/39 (2.5)	3/151 (1.9)	23/170 (13.5)	2/16 (12.5)	29/376 (7.7)
Respiratory rate	7/39 (17.9)	44/151 (29.1)	68/170 (40.0)	7/16 (43.7)	126/376 (33.5)
Heart rate	20/39 (51.3)	73/151 (48.3)	79/170 (46.5)	7/16 (43.7)	179/376 (47.6)
Oxygen saturation	36/39 (92.3)	127/151 (84.1)	102/170 (60.0)	7/16 (43.7)	272/376 (72.3)
Pulse oximetry should be done for					
Any patient in the health facility	29/39 (74.4)	80/151 (52.9)	67/170 (39.4)	11/16 (68.7)	187/376 (49.7)
Patient gasping for breath	1/39 (2.6)	14/151 (9.2)	14/170 (8.2)	1/16 (6.2)	30/376 (7.9)
Patient with emergency signs	3/39 (7.6)	16/151 (10.6)	20/170 (11.7)	1/16 (6.2)	40/376 (10.6)
Patient with respiratory symptoms	6/39 (15.3)	41/151 (27.1)	57/170 (33.5)	3/16 (18.7)	107/376 (28.4)
Patients with fever	0	0	5/170 (2.9)	0	5/376 (1.3)
Normal oxygen saturation (%) is					
80–100	2/39 (5.1)	13/151 (8.6)	40/170 (23.5)	0	55/376 (14.6)
85–100	1/39 (2.6)	9/151 (5.9)	10/170 (5.8)	1/16 (6.2)	21/376 (5.6)
90–100	6/39 (15.4)	38/151 (25.2)	45/170 (26.5)	5/16 (31.2)	94/376 (25.0)
95–100	30/39 (76.9)	90/151 (59.6)	62/170 (36.5)	7/16 (43.7)	189/376 (50.2)
WHO recommends oxygen therapy if oxygen saturation (SpO_2_) is less than (%)					
100	0	0	4/170 (2.3)	1/16 (6.2)	5/376 (1.3)
95	20/39 (51.2)	35/151 (23.2)	25/170 (14.7)	1/16 (6.2)	81/376 (21.5)
90	12/39 (30.7)	44/151 (29.1)	46/170 (27.1)	4/16 (25.0)	106/376 (28.2)
85	0	20/151 (13.2)	14/170 (8.2)	1/16 (6.2)	35/376 (9.3)
80	6/39 (15.4)	48/151 (31.8)	61/170 (35.8)	4/16 (25.0)	119/376 (31.6)
Good knowledge of pulse oximetry (4/4)	7/40 (17.5)	16/155 (10.3)	7/242 (2.9)	1/26 (3.8)	31/463 (6.7)

Others: health attendants and pharmacy technicians.

Overall, 6.7% had good pulse oximetry knowledge. Doctors had 6.11 times higher odds of having good pulse oximetry knowledge compared to other HCWs (CI: 2.05–18.16). Compared to those with no prior training experience, the odds of having good knowledge of pulse oximetry were two times higher among those with prior training within one year (2.0, CI: 0.79–5.04) and 1.44 times higher among those with training for than one year (1.44: 0.51–4.07) (Appendix 6).

### IMCI knowledge

Regarding IMCI knowledge, knowledge of danger signs was inconsistent. While convulsion (93.5%), lethargy (80.9%) and loss of consciousness (84.6%) were often correctly reported as general danger signs, a considerable proportion of HCWs incorrectly reported other clinical features like fever (doctors, 77.5%; nurses, 81.9%; CHWs, 78.5%; other 88.5%), cough (doctors, 47.5%; nurses, 67.7%; CHWs, 59.9%; others, 46.1%) and loss of appetite as general danger signs (doctors, 65%; nurses, 58.1%; CHWs, 51.2%; others, 46.1%). Moreover, knowledge of vomiting everything and being unable to eat/drink was low (Table 2). Out of the maximum score of eight, the mean knowledge score for general danger signs was higher among doctors (4.5 ± 1.4) compared with nurses (3.7 ± 1.5) and CHW (3.5 ± 1.4) (*p* < 0.05).

**Table 2:** Knowledge of danger signs among healthcare workers.

Receive training on:	Doctors (*N* = 40) Frequency (%)	Nurses (*N* = 155) Frequency (%)	CHEW/CHO (*N* = 242) Frequency (%)	Others (*N* = 26) Frequency (%)	Total (463)
General danger signs in children 2–59 months include					
Fever	31/40 (77.5)	127/155 (81.9)	190/242 (78.5)	23/26 (88.5)	371/463 (80.1)
Cough	19/40 (47.5)	105/155 (67.7)	145/242 (59.9)	12/26 (46.1)	281/463 (60.7)
Convulsion	40/40 (100.0)	144/155 (92.9)	224/242 (92.5)	25/26 (96.1)	433/463 (93.5)
Lethargy	38/40 (95.0)	130/155 (83.9)	186/242 (76.8)	21/26 (80.7)	375/463 (80.9)
Loss of appetite	26/40 (65.0)	90/155 (58.1)	124/242 (51.2)	12/26 (46.1)	252/463 (54.4)
Loss of consciousness	40/40 (100.0)	137/155 (88.3)	201/242 (83.1)	22/26 (84.6)	178/463 (38.4)
According to IMCI					
Vomiting severely and vomiting everything mean the same thing					
Yes	10/40 (25.0)	60/155 (38.7)	143/242 (59.1)	15/26 (57.7)	228/463 (49.2)
No	29/40 (72.5)	86/155 (55.5)	91/242 (37.6)	9/26 (34.6)	215/463 (46.4)
Loss of appetite and being unable to eat/drink mean the same thing					
Yes	9/40 (22.5)	80/155 (51.6)	144/242 (59.5)	19/26 (73.1)	252/463 (54.4)
No	31/40 (77.5)	71/155 (45.8)	90/242 (37.2)	5/26 (19.2)	197/463 (42.5)
Mean score (danger signs)*	4.5 ± 1.4	3.7 ± 1.5	3.5 ± 1.4	3.3 ± 1.6	3.6 ± 1.4

*p < 0.05. Others: health attendants and pharmacy technicians.

Less than 6% of the HCWs knew the four major IMCI symptoms that should be assessed in every child aged 2–59 months presenting at health facilities. For pneumonia, 55.7% of HCW correctly reported fast breathing and chest indrawing as any two symptoms required for pneumonia diagnosis in a child with cough and/or difficulty in breathing. Regarding pneumonia treatment, 87.5% of doctors indicated antibiotics (good knowledge), compared with 47.7% of nurses and 47.5% of CHWs who indicated antibiotics and cough syrup (bad knowledge). For signs of dehydration in a child with diarrhoea, 62.5% of doctors correctly picked sunken eyes, feeling thirsty and drinking eagerly compared to 40.0% of nurses and 34.3% of CHWs. The mean knowledge score (diagnosis and treatment) was significantly higher for doctors (7.8 ± 1.9) than nurses (6.6 ± 2.3) and CHWs (5.9 ± 2.4) (*p* < 0.05) (Table 3).

**Table 3:** Knowledge of integrated management of childhood illnesses guidelines (case management) among healthcare workers.

	Doctors (*N* = 40) Frequency (%)	Nurses (*N* = 155) Frequency (%)	CHEW/CHO (*N* = 242) Frequency (%)	Others (*N* = 26) Frequency (%)	Total (463)
Healthcare workers should ask for the following four symptoms in every child aged 2–59 months					
Cough or difficulty in breathing, diarrhoea, fever, ear pain	2/40 (5.0)	12/155 (7.7)	13/242 (5.4)	0	27/463 (5.8)
Danger signs, cough, malnutrition, and vaccine status	34/40 (85.0)	86/155 (55.5)	144/242 (59.5)	18/26 (69.2)	282/463 (60.9)
Fever, diarrhoea, vomiting, loss of appetite	4/40 (10.0)	28/155 (18.1)	32/242 (13.2)	3/26 (11.5)	67/463 (14.5)
Malnutrition, fever, cough, and danger signs	0	23/155 (14.8)	49/242 (20.2)	3/26 (11.5)	75/463 (16.2)
According to IMCI, a child with a cough/or difficulty breathing has PNEUMONIA if s/he has the following two additional symptoms/signs.					
Fever	20/40 (50.0)	49/155 (31.6)	74/242 (30.6)	13/26 (50.0)	156/463 (33.6)
Convulsion	1/40 (2.5)	13/155 (8.3)	37/242 (15.2)	2/26 (7.6)	67/463 (14.5)
fast breathing	34/40 (85.0)	104/155 (67.1)	174/242 (71.9)	15/26 (57.6)	327/463 (70.6)
Chest indrawing	26/40 (65.0)	107/155 (69.0)	142/242 (58.7)	7/26 (26.9)	282/463 (60.9)
Fast breathing and chest indrawing	20/40 (50.0)	81/155 (52.3)	138/242 (57.0)	19/26 (73.1)	258/463 (55.7)
A child with pneumonia should be given					
Antibiotics	35/40 (87.5)	71/155 (45.8)	101/242 (41.7)	15/26 (57.6)	222/463 (47.9)
Antibiotics and antimalaria	0	6/155 (3.8)	19/242 (7.8)	1/26 (3.8)	26/463 (5.6)
Antibiotics and cough syrup	5/40 (12.5)	74/155 (47.7)	115/242 (47.5)	10/26 (38.4)	204/463 (44.1)
Antimalaria	0	0	2/242 (0.8)	0	2/463 (0.4)
Cough syrup	0	1/155 (0.6)	1/242 (0.4)	0	2/463 (0.4)
According to IMCI, a child has malaria if the child has					
Fever and positive malaria test	29/40 (72.5)	88/155 (56.8)	116/242 (47.9)	9/26 (34.6)	242/463 (52.2)
Positive malaria diagnostic test	10/40 (25.0)	48/155 (30.9)	91/242 (37.6)	17/26 (65.3)	166/463 (35.8)
History of fever	0	8/155 (5.2)	14/242 (5.7)	0	22/463 (4.7)
A child with malaria should be given					
Antimalaria	35/40 (87.5)	103/155 (66.4)	158/242 (65.2)	21/26 (80.7)	317/463 (68.5)
Antimalarial and antibiotic	5/40 (12.5)	50/155 (32.3)	75/242 (30.9)	5/26 (19.2)	135/463 (29.2)
Antibiotics	0	0	1/242 (0.4)	0	1/463 (0.2)
Signs of dehydration in a child with diarrhoea include					
Fever	7/40 (17.5)	36/155 (23.2)	48/242 (19.8)	7/26 (26.9)	98/463 (21.1)
Flat abdomen	8/40 (20.0)	53/155 (34.2)	72/242 (29.7)	12/26 (80.7)	145/463 (31.3)
Sunken eyes	34/40 (85.0)	133/155 (85.8)	185/242 (76.4)	22/26 (84.6)	374/463 (80.7)
Feeling thirsty	32/40 (80.0)	111/155 (71.6)	144/242 (59.5)	20//26 (76.9)	307/463 (66.3)
Drinking eagerly	26/40 (65.0)	74/155 (47.7)	108/242 (44.6)	17//26 (65.3)	225/463 (48.5)
Tolerating small food	1/40 (2.5)	20/155 (12.9)	31/242 (12.8)	3/26 (11.5)	/463 (68.5)
Selected all three correct responses	25/40 (62.5)	62/155 (40.0)	83/242 (34.3)	16/26 (61.5)	186/463 (40.1)
A child with diarrhoea should be given					
Administer intravenous fluid	6/40 (15.0)	49/155 (31.6)	47/242 (19.4)	6/26 (23.1)	108/463 (23.3)
To give extra fluid at home	25/40 (62.5)	84/155 (54.2)	120/242 (49.5)	20/26 (76.9)	249/463 (53.8)
Advise the mother to do nothing the diarrhoea will stop	1/40 (2.5)	11/155 (7.1)	23/242 (9.5)	5/26 (19.2)	40/463 (8.6)
Should give the child antibiotic	6/40 (15.0)	44/155 (28.4)	56/242 (23.1)	7/26 (26.9)	113/463 (24.4)
Recommend the child should take zinc tablet	29/40 (72.5)	104/155 (67.1)	145/242 (59.9)	15/26 (57.6.)	249/463 (53.8)
Mean score (diagnosis and treatment)*	7.8 ± 1.9	6.6 ± 2.3	5.9 ± 2.4	6.1 ± 1.2	6.3 ± 2.4
Mean score (IMCI guideline)*	12.3 ± 2.4	10.3 ± 3.2	9.4 ± 3.1	9.5 ± 2.0	10 ± 3.1

*p < 0.05. Others: health attendants and pharmacy technicians.

## Discussion

We assessed the knowledge of pulse oximetry, oxygen saturation cut-off for oxygen therapy IMCI knowledge among HCWs in PHCs and secondary hospitals in Nigeria. Our study found inequality in HCWs’ knowledge about pulse oximetry. Despite national and global policies promoting pulse oximetry and IMCI as essential child survival strategies (9, 12, 23), we found a general low knowledge of pulse oximetry and oxygen therapy. Knowledge of general danger signs was low, with HCWs commonly identifying danger signs like convulsions and loss of consciousness, but also incorrectly including milder symptoms like fever and cough. Knowledge of key IMCI concepts was also low, and the use of cough syrup for pneumonia treatment was common among non-medical HCWs. These findings highlight missed opportunities in routine IMCI implementation and ongoing oxygen scale-up efforts. Given that IMCI has been adopted in Nigeria since 1997 (28) and its implementation has been constrained by longstanding challenges, including inadequate and unsustained government funding (3, 29), our findings likely reflect the cumulative effects of these systemic weaknesses. Recent shift towards pulse oximetry and oxygen therapy may further worsen IMCI implementation (12, 30, 31), but it also provides opportunities for health system strengthening, including IMCI implementation, if oxygen improvement efforts employ multi-prong strategies rather than vertical oxygen programmes.

In our study, approximately 50% of the HCWs knew pulse oximetry should be done for all patients, but just 28% knew the WHO threshold to commence oxygen therapy. This gap is concerning, as the correct interpretation of pulse oximetry readings is critical to identifying severely ill children. In this study, the proportion of doctors and nurses who reported in-service training on pulse oximetry/oxygen is higher than in a previous study conducted among Nigerian HCWs (doctors, 12.3%; nurses, 16.9%) (32). These findings show progress in pulse oximetry and oxygen scale-up efforts in Nigeria (13, 33), but also highlight a critical implementation gap and opportunity for improvement in scale-up efforts. Pulse oximetry is a useful triage tool to identify sick children with the highest risk of death (34, 35); therefore, scale-up efforts should emphasize correct interpretation of pulse oximetry readings and corresponding clinical actions. Given that previous studies of pulse oximetry introduction into routine healthcare service delivery in Nigeria have reported mixed results (10, 36), with varying mechanisms and barriers to adoption, partnerships with professional associations, inclusion of pulse oximetry in continuous professional development programs, and the use of online learning platforms may enhance knowledge and adoption among HCWs. Future studies can therefore explore the effectiveness of these methods for pulse oximetry scale-up.

We found low knowledge of IMCI general danger signs, which is similar to findings from previous studies within and outside Nigeria (37, 38). One of the explanations for our finding is the haphazard implementation of IMCI in Nigeria despite its long adoption (3). Most efforts to implement IMCI in Nigeria have been donor-driven, with limited success in terms of integration and sustainability (11). Anecdotally, there are also myths and misconceptions about IMCI in Nigeria, with some perceptions that IMCI is for non-medical personnel and that it is primarily used in remote rural settings. These systemic barriers with fragmented healthcare delivery undermine effective IMCI implementation. The National strategy on child survival has prioritised scale-up of IMCI as one of the key pillars to reducing under-five deaths (7), but sustainable adoption requires addressing these systemic barriers and misconceptions through inclusive collaboration among policymakers, trainers, frontline workers, and communities. Concerted efforts are required to strengthen pre-service training and skills acquisition on IMCI implementation in real-life settings. This requires alignment of other national policy documents with IMCI, like the national standing order for community health workers and national guidelines on pneumonia management, to avoid conflicting recommendations and promote IMCI integration in service delivery (39, 40).

In the past decades, there have been several innovations to expand the IMCI scope and utilisation in many low-resource settings (41). Electronic or digitalizd IMCI has been implemented in Tanzania, Uganda, Somalia, and Nigeria (42–44). Similarly, information, communication technology (ICT) has been used to enhance IMCI training (41),such as distance learning IMCI training in Tanzania, and android-based training piloted in Uganda (45, 46). Other interventions have expanded the IMCI scope to include HIV management and pulse oximetry screening (47, 48). However, despite these innovations being promising, results have been mixed, and some have been implemented within a short period of time. Adopting any of these approaches for future IMCI scale-up efforts in Nigeria requires further implementation research to understand sustainable and effective IMCI implementation strategies.

Finally, most of the HCWs in our studies have not been trained on Emergency Triage Assessment and Treatment, unlike Infection Prevention and Control. While this is not surprising, considering the recent COVID-19 pandemic and the huge burden of infectious diseases in Nigeria, this gap likely contributes to preventable deaths and highlights the need for prioritisation of emergency care services delivery to improve child survival (49). Previous studies in Nigeria have also documented similar findings (49–51). A narrative review of pediatric emergency medicine in Nigeria found that HCWs lack formal certification in pediatric emergency care. Another study among primary HCWs in Nigeria found that nearly half of the surveyed HCWs demonstrated poor knowledge of emergency skills and lacked appropriate treatment skills (52). These findings call for prioritisation of pediatric emergency care services, including widespread ETAT training, to strengthen Nigeria’s pediatric emergency system and reduce avoidable mortality.

Our study has two key limitations. Firstly, we used convenience sampling to select HCWs. It is possible that this approach introduced a selection bias; however, data collectors made return visits to capture HCWs that might have been missed during the previous visits. Secondly, knowledge was based on self-reported data from HCWs, and we did not conduct clinical observations to assess practice or conduct HCW interviews to explore motivations for their decision regarding pulse oximetry and IMCI adherence practice. We therefore cannot comment on the quality of care.

## Data Availability

The raw data supporting the conclusions of this article will be made available by the authors, without undue reservation.
