# Supplementary material for: Knowledge of pulse oximetry, indications for oxygen therapy, and integrated management of childhood illness among health care workers in Nigerian primary and secondary health facilities: a cross-sectional survey
Source: Front Public Health. 2026 Jul 8;14:1789259. doi: 10.3389/fpubh.2026.1789259 (PMC13388471; doi:10.3389/fpubh.2026.1789259)
Supplement: Supplementary file 1 [file Data_Sheet_1.ZIP › Submitted appendices/Appendix 5 HCW training experience.docx]

Appendix 3: Healthcare workers' training experiences.
