# Supplementary material for: Knowledge of pulse oximetry, indications for oxygen therapy, and integrated management of childhood illness among health care workers in Nigerian primary and secondary health facilities: a cross-sectional survey
Source: Front Public Health. 2026 Jul 8;14:1789259. doi: 10.3389/fpubh.2026.1789259 (PMC13388471; doi:10.3389/fpubh.2026.1789259)
Supplement: Supplementary file 1 [file Data_Sheet_1.ZIP › Submitted appendices/Appendix 1 State characteristics_.docx]

Appendix 4

| **States** | **Number of selected LGAs per state** | | |
| --- | --- | --- | --- |
| Jigawa | 3 | | |
| Kano | 4 | | |
| Lagos | 1 | | |
| Oyo | 5 | | |
| Rivers | 4 | | |
| **States** | **Geopolitical zones** | | |
| Jigawa | North-west | | |
| Kano | North-west | | |
| Lagos | South-west | | |
| Oyo | South-west | | |
| Rivers | South-south | | |
| **States** | **Under-five mortality rates *** | | |
| Jigawa | 174 | | |
| Kano | 148 | | |
| Lagos | 15 | | |
| Oyo | 57 | | |
| Rivers | 100 | | |
| **State** | **Primary** | **Secondary** | **Total** |
| **Number of facilities included in each state** | **54** | **8** | **62** |
| Jigawa | 8 | 3 | 11 |
| Kano | 6 | 2 | 8 |
| Lagos | 10 | 0 | 11 |
| Oyo | 20 | 3 | 23 |
| Rivers | 9 | 0 | 9 |
| **Overall responses in each state by type of facilities (Consented)** | **486** | **167** | **653** |
| Jigawa | 37 | 68 | 105 |
| Kano | 83 | 66 | 149 |
| Lagos | 124 | 0 | 124 |
| Oyo | 131 | 33 | 164 |
| Rivers | 111 | 0 | 111 |
| **Eligible Responses in each state by type of facilities (N=463)** | **324** | **139** | **463** |
| Jigawa | 31 | 65 | 96 |
| Kano | 59 | 48 | 107 |
| Lagos | 90 | 0 | 90 |
| Oyo | 88 | 26 | 114 |
| Rivers | 56 | 0 | 56 |

*Deaths per 1,000 live births
