# Supplementary material for: Knowledge of pulse oximetry, indications for oxygen therapy, and integrated management of childhood illness among health care workers in Nigerian primary and secondary health facilities: a cross-sectional survey
Source: Front Public Health. 2026 Jul 8;14:1789259. doi: 10.3389/fpubh.2026.1789259 (PMC13388471; doi:10.3389/fpubh.2026.1789259)
Supplement: Supplementary file 1 [file Data_Sheet_1.ZIP › Submitted appendices/Appendix 2 Pulse oxi scoring.docx]

|  |
| --- |
| **Know a pulse oximeter** |
| **A pulse oximeter is used for measuring oxygen saturation levels.** |
| Blood pressure |
| Respiratory rate |
| Heart rate |
| Oxygen saturation* |
| **Pulse oximetry should be done for** |
| Any patients in the health facility* |
| Patient gasping for breath |
| Patient with emergency signs |
| Patient with respiratory symptoms |
| Patients with fever |
| **Normal oxygen saturation (%) is** |
| 80-100 |
| 85-100 |
| 90-100 |
| 95-100* |
| **WHO recommends oxygen therapy if oxygen saturation (SpO2) is less than (%)** |
| 100 |
| 95 |
| 90* |
| 85 |
| 80 |

**Pulse oximetry scoring**

The correct option has an asterisk (*)
