# Supplementary material for: Knowledge of pulse oximetry, indications for oxygen therapy, and integrated management of childhood illness among health care workers in Nigerian primary and secondary health facilities: a cross-sectional survey
Source: Front Public Health. 2026 Jul 8;14:1789259. doi: 10.3389/fpubh.2026.1789259 (PMC13388471; doi:10.3389/fpubh.2026.1789259)
Supplement: Supplementary file 1 [file Data_Sheet_1.ZIP › Submitted appendices/Appendix 4_sociodemographics.docx]

| **Variables** | **Primary**  **(n=324)** | **Secondary**  **(n=139)** | **Total (%)**  **(N=463)** |
| --- | --- | --- | --- |
| **Gender** |  |  |  |
| Male | 65/324(20.1) | 49/139(35.3) | 114 / 463 (24.6) |
| Female | 259/324(79.9) | 90/139(64.7) | 349/ 463 (75.4) |
| **Age (in years)** | **311** | **129** | **440** |
| 16-29 | 88/311(28.3) | 49/129(38.0) | 137/440 (31.1) |
| 30-49 | 194/311(62.4) | 64/129(49.6) | 258/440 (58.7) |
| ≥50 | 29/311(9.3) | 16/129(12.4) | 45/440(10.2) |
| **Level of education** |  |  |  |
| Primary | 2/324(0.6) | 0 | 2 / 463 (0.4) |
| Secondary | 1/324(0.3) | 3/139(2.1) | 4/ 463 (0.9) |
| Tertiary | 284/324(87.7) | 111/139(79.9) | 395 / 463 (85.3) |
| Postgraduate | 37/324(11.4) | 25/139(18.0) | 62/ 463 (13.4) |
| **Cadre** |  |  |  |
| Doctor | 22/324(6.8) | 18/139(12.9) | 40 / 463 (8.6) |
| Nurse(midwifery) | 74/324(22.8) | 81/139(58.3) | 155/ 463 (33.5) |
| CHEW/CHO | 204/324(63.0) | 38/139(27.3) | 242 / 463 (52.3) |
| Health assistants/pharmacy technicians | 24/324(7.4) | 2/139(1.4) | 26/ 463 (5.6) |
| **Employment type*** | **323** | **139** | **462** |
| Government employed | 232/323(71.8) | 109/139(78.4) | 341/462 (73.8) |
| Facility employed (e.g, ad hoc staff) | 39/323 (12.1) | 5/139(3.6) | 44/462(9.5) |
| Volunteer | 52/323(16.1) | 25/139 (18.0) | 77/462(16.7) |
| **State** |  |  |  |
| Jigawa | 31/324(9.6) | 65/139(46.8) | 96 / 463 (20.7) |
| Kano | 59/324(18.2) | 48/139(34.5) | 107/ 463 (23.1) |
| Lagos | 90/324 (27.8) | 0 | 90 / 463 (19.4) |
| Oyo | 88/324 (27.2) | 26/139(18.7) | 114/463 (24.6) |
| Rivers | 56/324 (17.2) | 0 | 56/463 (12.1) |

*Appendix 4 Sociodemographic characteristics of respondents*
