# Supplementary material for: Knowledge of pulse oximetry, indications for oxygen therapy, and integrated management of childhood illness among health care workers in Nigerian primary and secondary health facilities: a cross-sectional survey
Source: Front Public Health. 2026 Jul 8;14:1789259. doi: 10.3389/fpubh.2026.1789259 (PMC13388471; doi:10.3389/fpubh.2026.1789259)
Supplement: Supplementary file 1 [file Data_Sheet_1.ZIP › Submitted appendices/Appendix 6_poX Predictors_28 APril.docx]

Appendix 6 Predictors of pulse oximetry knowledge

| **Variables** | **Knowledge of pulse oximetry** | | **Crude Odds ratio (95% CI)** | **Adjusted odds ratio (95% CI)** |
| --- | --- | --- | --- | --- |
|  | **Good (n=31)** | **Poor (n=432)** |  |  |
| **State** |  |  |  |  |
| Lagos (n=90) | 12/90 (13.33) | 78/90 (86.67) | **7.23 (1.57 to 33.28)** |  |
| Oyo (n=114) | 9/114(7.89) | 105/114(92.11) | 4.03(0.84 to 19.11) |  |
| Rivers (n=56) | 6/56 (10.71) | 50/56 (89.29) | **5.63 (1.09 to 28.97)** |  |
| Kano(n=107) | 2/107 (1.87) | 105/107 (98.13) | **0.89 (0.12 to 6.48)** |  |
| Jigawa (n=96) | 2/96 (2.08) | 94/96 (97.92) | reference |  |
| **Health workers cadre** |  |  |  |  |
| Doctors (n=40) | 7/40 (17.50) | 33/40 (82.50) | **6.89 (2.34 to 20.24)** | **5.26 (1.76 to 15.78)** |
| Nurses (n=155) | 16/155(10.32) | 139/155(89.68) | **3.74(1.56 to 8.95)** | **4.34 (1.77 to 10.68)** |
| Others (n=268) | 8/268 (2.89) | 260/268 (97.01) | Reference |  |
| **Type of facility** |  |  |  |  |
| Primary (n=324) | 24/324(7.40) | 300/324 (92.50) | 1.5 (0.63 to 3.59) | 0.35 (0.10 to 1.17) |
| Secondary (n=139) | 7/139 (5.00) | 132/139 (95.0) | Reference |  |
| **Past Training on the pulse oximeter** |  |  |  |  |
| Yes, within one year (n=69) | 7/69 (10.14) | 62/69 (89.86) | 1.81(0.73 to 4.49) | 1.75 (0.68 to 4.50) |
| Yes, more than one year (n=70) | 5/70 (7.14) | 65/70 (92.86) | 1.23(0.44 to 3.43) | 1.03 (0.35 to 3.06) |
| No (n=324) | 19/324(5.86) | 305/324(94.14) | Reference |  |

**Bold figures** =significant at p<0.05
